# The Effect of Inoculum Size on Antimicrobial Susceptibility Testing of Mycobacterium tuberculosis

**DOI:** 10.1128/spectrum.00319-23

**Published:** 2023-05-22

**Authors:** Kubra Yildirim, Cemilenur Atas, Ece Simsek, Ahmet Yilmaz Coban

**Affiliations:** a Akdeniz University Tuberculosis Research Center, Antalya, Turkey; b Akdeniz University, Faculty of Health Sciences, Department of Nutrition and Dietetics, Antalya, Turkey; c Akdeniz University Institute of Health Sciences, Department of Medical Biotechnology, Antalya, Turkey; University of California, San Diego

**Keywords:** *Mycobacterium tuberculosis*, inoculum effect, drug susceptibility test, inoculum size

## Abstract

Phenotypic drug susceptibility testing (DST) requires a standardized amount of inoculum to produce reproducible susceptibility results. The most critical step in the application of DST in Mycobacterium tuberculosis isolates is the preparation of the bacterial inoculum. In this study, the effect of bacterial inoculum prepared in various McFarland turbidities on primary antituberculosis drug susceptibility of M. tuberculosis strains was investigated. Five standard ATCC strains (ATCC 27294 [H37Rv], ATCC 35822 [izoniazid-resistant], ATCC 35838 [rifampicin-resistant], ATCC 35820 [streptomycin-resistant], ATCC 35837 [ethambutol-resistant]) were tested. Inoculums of McFarland standard of 0.5, 1, 2, 3, and 1:100 dilutions of 1 McFarland standard of each strain were used. The effect of inoculum size on DST results was determined by the proportion method in Lowenstein-Jensen (LJ) medium and nitrate reductase assay (NRA) in the LJ medium. In both test methods, the increase in inoculum size did not affect the DST results of the strains. On the contrary, DST results were obtained more rapidly as a result of the use of dense inoculum. DST results obtained in all McFarland turbidities were found to be 100% compatible with the recommended amount of inoculum, 1:100 dilution of 1 McFarland standard (inoculum size of gold standard method). In conclusion, the use of a high amount of inoculum did not change the drug susceptibility profile of tuberculosis bacilli. Minimizing manipulations during the inoculum preparation phase of susceptibility testing, this outcome will decrease the need for equipment and make the test application easier, particularly in developing countries.

**IMPORTANCE** During DST application, it can be challenging to evenly homogenize TB cell clumps with lipid-rich cell walls. These experiments must be carried out under Biosafety Level-3 (BSL-3) laboratory conditions with personal protective equipment and taking safety precautions because the procedures applied at this stage cause the formation of bacillus-laden aerosols and carry a serious risk of transmission. Considering this situation, this stage is important given that it is not possible to establish a BSL-3 laboratory in poor and developing countries. Reducing the manipulations to be applied during the preparation of bacterial turbidity will minimize the risk of aerosol formation. Perhaps there will be no need to do these steps for susceptibility tests in these countries or even in developed countries.

## INTRODUCTION

Tuberculosis (TB) is in second place after COVID-19 in terms of deaths due to a single infectious agent (1). Multidrug-resistance (MDR) and extensively drug-resistance (XDR) in Mycobacterium tuberculosis isolates pose a significant challenge for TB control programs (2). Culture-based phenotypic drug susceptibility testing (DST) is currently the gold standard for the detection of drug resistance. The phenotypic DST for M. tuberculosis is based on testing a single critical concentration that is specific for each anti-TB agent and test method (3). The most critical step in the application of DST in M. tuberculosis isolates is the preparation of mycobacterial inoculum. Clumping of TB bacilli causes uncertainty in the bacillus distribution in the inoculum and the number of bacilli per milliliter. This is a critical step in terms of test reproducibility (4). Phenotypic DST requires a standardized amount of inoculum to produce reproducible susceptibility results. In the documents published by the Clinical and Laboratory Standards Institute (CLSI) in TB susceptibility tests, it is recommended to use the inoculum between McFarland standard of 0.5 to 1 (5–7). In Etest studies, it is recommended to use a McFarland standard of ≥3 bacterial density (8). During DST application, it can be challenging to evenly homogenize TB cell clumps with lipid-rich cell walls. These experiments must be carried out under Biosafety Level-3 (BSL-3) laboratory conditions with personal protective equipment and taking safety precautions because the procedures applied at this stage cause the formation of bacillus-laden aerosols and carry a serious risk of transmission (9, 10). Considering this situation, this stage is important given that it is not possible to establish a BSL-3 laboratory in poor and developing countries.

The number, distribution, and viability of organisms in the inoculum have a significant impact on DST results. To obtain reliable DST results, the inoculum preparation must be properly standardized (11). However, there are not enough studies on the effect of inoculum size on the drug susceptibility of M. tuberculosis strains. It has been previously shown that the inoculum size of 0.1 to 3 mg does not affect the streptomycin (STR) susceptibility of M. tuberculosis. However, experimental study data are insufficient (12).

However, resistance in M. tuberculosis isolates is usually due to spontaneous mutations. Therefore, the denser the amount of inoculum, the easier it will be to detect resistant isolates in susceptibility testing (13). For this purpose, the effects of bacterial inoculums with various McFarland turbidities on susceptibility tests were investigated in Löwenstein-Jensen (LJ) medium with standard ATCC strains using both the proportion method and nitrate reductase assay (NRA).

## RESULTS

In the study, the tests for all bacterial densities were completed on the 21st day for the proportion method in the LJ medium. DST results with inoculum sizes used for all strains were 100% consistent with the recommended inoculum size (1:100 dilution of 1 McFarland standard inoculum) (Table 1). On the other hand, microcolonies were observed in 2 and 3 McFarland standard inoculums in LJ medium containing EMB in the ATCC-STR strain set. However, the number of these microcolonies was less than 1% of the number of colonies in the control tubes. Therefore, the categorical agreement of the strain remained the same. In the NRA, susceptibility results for all inoculum sizes, including the recommended inoculum size, were 100% consistent. DST results obtained with NRA are shown in Table 2. For the H37Rv strain, NRA results for the McFarland standard of 0.5, 1, 2, and 3 inoculums were obtained on the 5th day of incubation. Results for 1/100 dilution of 1 McFarland standard were obtained on the 10th day of incubation. For ATCC 35822 (INH-resistant) strain, NRA results were obtained for the 2 and 3 McFarland standard inoculums on the 7th day, 1 McFarland standard on the 10th day, 0.5 McFarland standard on the 14th day, 1/100 dilution of 1 McFarland standard on the 21st day of incubation. For ATCC 35838 (RIF-resistant) strain, NRA results were obtained for the 0.5, 1, 2, and 3 McFarland standard inoculums on the 7th day, 1:100 dilution of 1 McFarland standard on the 14th day of incubation. For ATCC 35820 (STR-Resistant) strain, NRA results were obtained for the 1, 2, and 3 McFarland standard inoculums on the 5th day, 0.5 McFarland standard on the 7th day, 1:100 dilution of 1 McFarland standard on the 10th day of incubation. For ATCC 35837 (EMB-Resistant) strain, NRA results were obtained for the 3 McFarland standard inoculums on the 7th day, 0.5, 1, and 2 McFarland standard on the 10th day, 1:100 dilution of 1 McFarland standard on the 14th day of incubation (Table 3).

**TABLE 1:** The effect of inoculum size on primary antituberculosis drug susceptibility of M. tuberculosis strains by proportion method on the LJ medium^*a*^

M. tuberculosis	McFarland no	Primary antibiotics			
		INH	RIF	STR	EMB
ATCC 27294 (H37Rv)	Standard inoculum	S	S	S	S
	0.5	S	S	S	S
	1	S	S	S	S
	2	S	S	S	S
	3	S	S	S	S
ATCC 35822 (INH-resistant)	Standard inoculum	R	S	S	S
	0.5	R	S	S	S
	1	R	S	S	S
	2	R	S	S	S
	3	R	S	S	S
ATCC 35838 (RIF-resistant)	Standard inoculum	S	R	S	S
	0.5	S	R	S	S
	1	S	R	S	S
	2	S	R	S	S
	3	S	R	S	S
ATCC 35820 (STR-resistant)	Standard inoculum	S	S	R	S
	0.5	S	S	R	S
	1	S	S	R	S
	2	S	S	R	S
	3	S	S	R	S
ATCC 35837 (EMB-resistant)	Standard inoculum	S	S	S	R
	0.5	S	S	S	R
	1	S	S	S	R
	2	S	S	S	R
	3	S	S	S	R

a R, resistant; S, susceptible; Standard inoculum, 1:100 dilution of 1 McFarland Standard.

**TABLE 2:** The effect of inoculum size on primary antituberculosis drug susceptibility of M. tuberculosis strains by NRA on the LJ medium^*a*^

M. tuberculosis	McFarland standard	Primary antibiotics			
		INH	RIF	STR	EMB
ATCC 27294 (H37Rv)	Standard inoculum	S	S	S	S
	0.5	S	S	S	S
	1	S	S	S	S
	2	S	S	S	S
	3	S	S	S	S
ATCC 35822 (INH-resistant)	Standard inoculum	R	S	S	S
	0.5	R	S	S	S
	1	R	S	S	S
	2	R	S	S	S
	3	R	S	S	S
ATCC 35838 (RIF-resistant)	Standard inoculum	S	R	S	S
	0.5	S	R	S	S
	1	S	R	S	S
	2	S	R	S	S
	3	S	R	S	S
ATCC 35820 (STR-resistant)	Standard inoculum	S	S	R	S
	0.5	S	S	R	S
	1	S	S	R	S
	2	S	S	R	S
	3	S	S	R	S
ATCC 35837 (EMB-resistant)	Standard inoculum	S	S	S	R
	0.5	S	S	S	R
	1	S	S	S	R
	2	S	S	S	R
	3	S	S	S	R

a R, resistant; S, susceptible; Standard inoculum, 1:100 dilution of 1 McFarland Standard.

**TABLE 3:** The effect of inoculum size of M. tuberculosis strains on the time required to determine drug susceptibility results with NRA

	Inoculum size (McFarland standard)				
M. tuberculosis	0.5	1	2	3	Standard inoculum
ATCC 27294 (H37Rv)	5th day	5th day	5th day	5th day	10th day
ATCC 35822 (INH-resistant)	14th day	10th day	7th day	7th day	21st day
ATCC 35838 (RIF-resistant)	7th day	7th day	7th day	7th day	14th day
ATCC 35820 (STR-resistant)	7th day	5th day	5th day	5th day	10th day
ATCC 35837 (EMB-resistant)	10th day	10th day	10th day	7th day	14th day

## DISCUSSION

Both World Health Organization (WHO) and CLSI, recommend 1:100 and 1:10,000 dilutions from 1 McFarland standard (CLSI previously recommended using McFarland standard of 0.5) as the amount of inoculum in susceptibility methods performed on both LJ medium and Middlebrook 7H10-11 agar (5–7). However, in the Etest susceptibility method, ≥3 McFarland standard inoculum is recommended (8).

There are also phenotypic colorimetric susceptibility testing methods. In these methods, there are differences between bacterial inoculums. In the NRA test performed on LJ medium, 0.2 mL of 1 McFarland standard turbidity is directly inoculated on antibiotic media. Also, 0.2 mL from 1:10 dilution of 1 McFarland standard was inoculated on growth control media (14, 15). In the NRA test performed directly from the clinical sample, the same dilution and amount of inoculum are used. (16, 17). In these studies, the number of bacteria inoculated on antibiotic media was used higher than in the control medium. When the NRA test is performed on microplates, a 1:10 dilution of 1 McFarland turbidity is used, and 100 μL was inoculated in each well of 96 microplates (18). In the Resazurin Microtiter Assay (REMA), 1:20 and 1:10 dilutions of 1 McFarland standard are used and 100 μL was inoculated in each well of 96 microplates (19, 20). In malachite green microtube assay (MGMT), 1:5 dilution of 1 McFarland standard (21, 22) and 1:10 dilution of 1 McFarland standard in crystal violet decolorization assay (CVDA) is used (23). As in these studies, there are differences between bacterial inoculum in the used methods.

Resistance in M. tuberculosis isolates depends on the presence of mutants. The average mutation rate for resistance to INH, RIF, EMB, and STR is 2.56 × 10^−8^, 2.25 × 10^−10^, 1 × 10^−7^, and 2.95 × 10^−8^, respectively (24). Therefore, the use of high-density inoculum may facilitate the detection of these mutants. However, the bacterial density used in the study was approximately 10^6^−6 × 10^6^ CFU/mL (McFarland no 0.5–3). These mutant isolates could not be detected because the tested bacterial density was far below the rate of resistant bacteria due to mutation. In other words, the number of bacterial inoculums to show false-drug resistance due to these mutants could not be reached.

Because TB bacilli tend to cluster together, it is difficult to count bacteria in liquid culture (25). In the study, the bacterial concentration in the inocula was determined as done previously by Peñuelas-Urquides et al. (25). The inocula were not plated to determine the bacterial concentration. However, we know from the literature how many bacteria the McFarland standard turbidities are equivalent to for M. tuberculosis. For example, M. tuberculosis H37Rv ATCC 27294 that 1 McFarland unit is equivalent to either 1.97× 10^6^ CFU/mL or 0.39 OD600, and an OD600 measurement of 1 is equivalent to either 3.13× 10^7^ CFU/mL or 3.66 McFarland units (25). Colony counting on agar plates is often used to determine the number of cells in a bacterial suspension. This requires an incubation period of at least 3 weeks. Although this method is time-consuming, it is not easily applicable to TB bacillus due to the uncertain growth of single cells. It is also difficult to make direct microscopic counts due to the clustering of TB bacilli and the inability to clearly distinguish between dead and live bacilli (26, 27). Considering these disadvantages, in the 1950s, bacillus weight per unit volume or nitrogen weight per unit volume were often used to determine the amount of TB bacillus (27). The relationship between the wet weight of the bacillus in the TB bacillus suspension and the turbidity of that suspension has been explained before and has allowed us to compare our study results with the limited existing studies in the literature. It has been reported that the weight of 1 mg of dry TB bacillus is equivalent to approximately 3.10^8^ TB bacillus with repetitions performed 6 times and considering that the bacilli contain 85% water, 1 mg of wet TB bacillus is equivalent to approximately 4.10^8^ bacilli (28).

It was shown by Youmans (29) that inoculum size between 0.1 and 1.0 mg did not affect the bacteriostatic activity of STR for TB bacillus. Additionally, 4 years later, Youmans and Williston together reported that 0.01 to 0.25 mg inoculum size did not affect the STR susceptibility test results (30). Previous studies were supported by showing that inoculum size between 0.1 and 3.0 mg, which is a larger inoculum range, does not affect the STR sensitivity of TB bacilli (12). Contrary to STR, it has been reported that there is an inverse relationship between the bacteriostatic effect of para-aminosalicylic acid (PAS) and the inoculum density of TB bacillus. Thus, drug susceptibility is affected by the number of bacteria in the inoculum (31). In addition, it has been reported that the size of the inoculum, which contains 2.5 to 3.7 mg of wet TB bacillus and its 10^−1^ to 10^−7^dilutions, affects the apparent sensitivities of STR, INH, and PAS (32). In summary, studies have shown that the STR susceptibility results of a minimum of 0.01 mg and a maximum of 3 mg of wet TB bacillus are the same and are not affected by the amount of inoculum.

Only STR, PAS, and INH were known as anti-TB drugs in the years 1945 to 1955 when the effect of the amount of inoculum on drug susceptibility results of TB bacilli was investigated. Other anti-TB drugs have not yet been discovered and their activities on TB have not been determined (33). Was the “inoculum effect,” a widely accepted laboratory phenomenon (34), also seen between primary anti-TB drugs and M. tuberculosis strains? Did the increase in inoculum density cause a significant increase in the MIC values of antibiotics? The results of our study based on these questions also show that there was no change in the DST results of TB bacilli from the recommended standard inoculum amount to 3 McFarland turbidity. However, although the generation time of bacilli was not affected as a result of using dense inoculum, DST results were obtained faster. Presumably, this allows earlier observation of bacterial communities formed due to the high bacterial count. To talk about the phenomenon of the “inoculum effect,” obviously higher inoculum amounts should be used. Jung et al. claimed that the problems experienced during the homogenization of TB bacillus clusters and the problems caused by unequal fragmentation will be reflected in the DST results, cause an increase in MICs, and therefore lead to the inoculum effect (35). However, no such effect was found in our study. This may be because MIC was not investigated in our study and only one critical concentration was used. Jung et al. (35) stated that there may be an increase in MIC values. However, if this increase does not affect the categorical group of susceptible or resistant strains, they cannot be determined by the proportion method.

The formation of microcolonies is another important result of our study. The formation of microcolonies already in the presence of EMB has been reported previously (36). However, it is difficult to decide whether these microcolonies represent resistant or partially resistant mycobacteria or an overgrowth of susceptible organisms following drug degradation (5). Because LJ medium is an egg-based medium, it must be heated at a high temperature to solidify. This situation may cause degradation and decrease in stability of antibiotics added to LJ medium for proportion method. Therefore, the critical concentrations of drugs vary in different media using the same method. For example, RIF is tested with the proportion method at a concentration of 1.0 μg/mL in 7H10 agar medium, while it is tested at a concentration of 40 μg/mL in LJ medium. Likewise, while STR is tested at a concentration of 2 μg/mL in 7H10 agar medium, it is tested at a concentration of 4 μg/mL in LJ medium. However, the same is not true for EMB. While it is used at a concentration of 5 μg/mL in 7H10 Agar medium, it is used at a concentration of 2 μg/mL in LJ medium. Inconsistent DST results may occur following the degradation of EMB, depending on factors such as the heating time and heating temperature of the LJ medium, as well as the difference between their critical concentrations. In this case, it can result in the formation of microcolonies in the presence of EMB. It has already been reported by the World Health Organization (WHO) that the phenotypic DST for EMB is not reliable and reproducible, with inconsistent drug susceptibility results (3).

Because resistance in M. tuberculosis isolates usually occurs due to spontaneous mutations, the higher the amount of inoculum, the easier it will be to detect resistant isolates in the susceptibility test. Furthermore, because the increase in bacterial density (high McFarland values as used in the Etest method) does not change the drug susceptibility profile of TB bacilli, the procedures to be applied during the preparation of the inoculum will be reduced and both the laboratory workload and the risk of transmission will be minimized.

As a result, TB is more common, especially in poor and developing countries and it is not possible to provide BSL-3 laboratory conditions in these countries. Reducing the manipulations to be applied during the preparation of bacterial turbidity will minimize the risk of aerosol formation. Perhaps there will be no need to do these steps for susceptibility tests in these countries or even in developed countries. Further studies are needed on this subject by increasing the number of clinical isolates.

## MATERIALS AND METHODS

### Bacterial strains.

In this study, strains of M. tuberculosis ATCC 27294 (H37Rv), ATCC 35822 (isoniazid [INH]-resistant), ATCC 35838 (rifampicin [RIF]-resistant), ATCC 35820 (streptomycin [STR]-resistant), ATCC35837 (ethambutol [EMB]-resistant) were tested.

### Preparation of bacterial inoculum.

During the preparation of bacterial inoculum, all experiments were carried out in a Class II type B microbiological safety cabinet in the BSL-3 laboratory, with personal protective equipment (3M Versaflo, TR-300+) taking safety precautions. Inoculums of bacterial strains were prepared using fresh cultures grown on LJ medium. After transferring colonies from fresh cultures to tubes containing eight to 10 sterile glass beads and 3 to 5 mL of saline, the tubes were tightly closed and wrapped with parafilm. Afterward, it was mixed at 2,500 rpm with a vortex (Onilab MX-S, City of industry, CA 91748 USA) for about 1 min, then the tubes were kept in an upright position at room temperature for about 30 to 60 min to precipitate aerosols and large particles. Then, the supernatant was transferred to another tube and the separated supernatant was adjusted to McFarland standard of 0.5, 1, 2, and 3 for each strain by the densitometer (BIOSAN Medical-Biological Research & Technologies, Riga, Latvia). In addition, for the recommend standard inoculum size, the McFarland standard of 1 was used to prepare 1:100 dilution (3).

### Preparation of antibiotic solutions.

In this study, primary antibiotics INH, RIF, STR, and EMB were tested. Powder forms of all antibiotics (Sigma-Aldrich) were used. INH, STR, and EMB were dissolved in sterile distilled water and RIF was dissolved in methanol. Stocks were prepared at a final concentration of 4,096 μg/mL for each antibiotic. Antibiotics were stored in aliquots at −80°C until use.

### Preparation of LJ medium for the proportion method.

LJ medium (Merck, Germany) was prepared according to the manufacturer's recommendations. After adding eggs, antibiotics were added to the medium, with final concentrations of 0.2 μg/mL, 40 μg/mL, 4 μg/mL, and 2 μg/mL for INH, RIF, STR, and EMB, respectively. Then, the 5 mL medium was dispensed into screw cap tubes. Similarly, antibiotic-free growth control tubes were also prepared. The tubes were heated at 85°C for 40 to 50 min in a slanted position. The prepared media were stored at +4°C. The storage period did not exceed 4 weeks (3).

### Preparation of LJ medium for nitrate reductase assay.

LJ medium was prepared as described above. In addition, KNO_3_ was added to the medium at a final concentration of 1,000 μg/mL. Antibiotic concentrations were the same as those used in the proportion method. For each strain, five antibiotic-free growth control tubes were also prepared.

### Proportion method on LJ medium.

**FIG 1:**
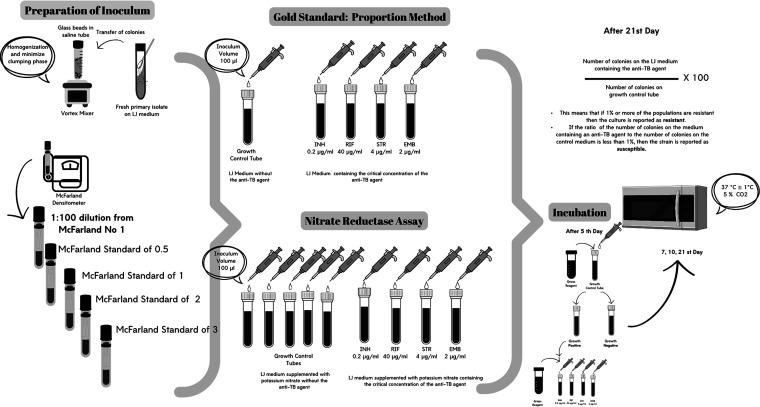
Test administration using the proportion method and nitrate reductase assay in LJ media for each strain.

The LJ proportion method is illustrated in Fig. 1. In the study, five sets were prepared for each strain. Each set consisted of a growth control and LJ media containing four primary anti-TB agents. Each set also contained McFarland standards of 0.5, 1, 2, 3, and 1:100 dilution of 1 McFarland standard inoculum. A 1:100 dilution of 1 McFarland standard inoculum was the recommended inoculum size. In addition, 100 μL of prepared inoculums for each strain were inoculated into LJ mediums with and without antibiotics (Fig. 1). LJ tubes were incubated at 37°C for 21 days. For contamination, it was checked every 3 days during the first week, then weekly. If sufficient growth was observed in the growth control tube at the end of the incubation, the test was terminated. The 1% proportion approach is used to evaluate the results. Accordingly, the culture is considered resistant if at least 1% of the population demonstrates resistance. The strain is classified as susceptible if the difference between the number of colonies on the anti-TB agent-containing medium and the number of colonies on the control medium is less than 1% (3).

### Nitrate reductase assay on LJ medium.

The NRA method is illustrated in Fig. 1. The inoculums prepared in this study were applied simultaneously for the NRA. Additionally, in the NRA, five antibiotic-free growth control tubes were used for each strain. And 100 μL of prepared inoculums for each strain were inoculated into LJ mediums with and without antibiotics (Fig. 1). After inoculation, the tubes were incubated at 37°C for 5, 7, 10, 14, and 21 days. After 5 days of incubation, 500 μL of Griess reagent (one part of 50% [vol/vol] concentrated hydrochloric acid, two parts of 0.2% [wt/vol] sulfanilamide, and two parts of 0.1% [wt/vol] n-1-naphthyl-ethylenediamine dihydrochloride) was added to one of the growth control tubes. If purple/violet color formation was observed in the growth control tube, 500 μL of Griess reagent was added to the antibiotic tubes, the test was terminated and the susceptibility results were evaluated. The susceptibility results were evaluated according to the color transformation in the growth control tube. If a purple/violet color appeared in the growth control tube after the addition of reagent in the tubes containing antibiotics, the isolate was considered resistant to the tested antibiotic. If no color was observed, the isolate was considered susceptible to the tested antibiotic. If purple/violet color formation was not observed in the growth control tube, no reagent was added to the antibiotic tubes, and incubation was continued. The same procedures were performed on the other growth control tubes on the 7th, 10th, 14th, and 21st days of incubation (14).

## References

[1] World Health Organization. 2022. Global tuberculosıs report 2022. World Health Organization, Geneva, Switzerland.

[2] Seung KJ, Keshavjee S, Rich ML. 2015. Multidrug-resistant tuberculosis and extensively drug-resistant tuberculosis. Cold Spring Harb Perspect Med 5:a017863. doi:10.1101/cshperspect.a017863. 25918181 PMC4561400

[3] World Health Organization. 2018. Technical manual for drug susceptibility testing of medicines used in the treatment of tuberculosis. World Health Organization, Geneva, Switzerland.

[4] Schön T, Werngren J, Machado D, Borroni E, Wijkander M, Lina G, Mouton J, Matuschek E, Kahlmeter G, Giske C, Santin M, Cirillo DM, Viveiros M, Cambau E. 2020. Antimicrobial susceptibility testing of Mycobacterium tuberculosis complex isolates- the EUCAST broth microdilution reference method for MIC determination. Clin Microbiol Infect 26:1488–1492. doi:10.1016/j.cmi.2020.07.036. 32750539

[5] Clinical and Laboratory Standards Institute. 2018. Susceptibility testing of Mycobacteria Nocardia spp., and other aerobic actinomycetes, 3rd ed. CLSI standard M24. CLSI, Wayne, PA. 31339680

[6] Clinical and Laboratory Standards Institute. 2011. Susceptibility testing of Mycobacteria, Nocardiae, and other aerobic actinomycetes; approved standard, 2nd ed. M24-A2. CLSI, Wayne, PA. 31339680

[7] National Committee for Clinical Laboratory Standards. 1995. Antimycobacterial susceptibility testing for Mycobacterium tuberculosis; tentative standard. M24-T. NCCLS, Wayne, PA.

[8] AB Biodisk. 2005. E-test technical booklet for Mycobacterium tuberculosis. AB Biodisk, Solna, Sweden.

[9] European Centre for Disease Prevention and Control. 2016. Handbook on TB laboratory diagnostic methods in the European Union. ECDC, Stockholm, Sweden.

[10] World Health Organization. 2020. Laboratory bıosafety manual, 4th ed. World Health Organization, Geneva, Switzerland.

[11] Kim SJ. 2005. Drug-susceptibility testing in tuberculosis: methods and reliability of results. Eur Respir J 25:564–569. doi:10.1183/09031936.05.00111304. 15738303

[12] Robinson JH, Cummings MM, Patnode RA. 1950. Comparison of two solid media for testing sensitivity to streptomycin. Am Rev Tuberc 62:484–490. 14790226 10.1164/art.1950.62.5.484

[13] McGrath M, Gey van Pittius NC, van Helden PD, Warren RM, Warner DF. 2014. Mutation rate and the emergence of drug resistance in Mycobacterium tuberculosis. J Antimicrob Chemother 69:292–302. doi:10.1093/jac/dkt364. 24072169

[14] Coban AY, Birinci A, Ekinci B, Durupinar B. 2004. Drug susceptibility testing of Mycobacterium tuberculosis with nitrate reductase assay. Int J Antimicrob Agents 24:304–306. doi:10.1016/j.ijantimicag.2004.02.027. 15325439

[15] Singh S, Kumar P, Sharma S, Mumbowa F, Martin A, Durier N. 2012. Rapid identification and drug susceptibility testing of Mycobacterium tuberculosis: standard operating procedure for non-commercial assays: part 2: nitrate reductase assay v1.3.12. J Lab Physicians 4:112–119. doi:10.4103/0974-2727.105593. 23440455 PMC3574495

[16] Mishra B, Muralidharan S, Srinivasa H. 2009. Direct drug susceptibility testing of Mycobacterium tuberculosis to primary anti-tubercular drugs by nitrate reductase assay. Indian J Pathol Microbiol 52:343–344. doi:10.4103/0377-4929.54989. 19679956

[17] Satti L, Ikram A, Palomino JC, Martin A, Khan FA. 2013. Field evaluation of the direct detection of multidrug-resistant Mycobacterium tuberculosis by nitrate reductase assay on 7H11 agar. Tuberculosis (Edinb) 93:308–311. doi:10.1016/j.tube.2013.02.013. 23507185

[18] Coban AY, Akbal AU, Uzun M, Durupinar B. 2015. Evaluation of four colorimetric susceptibility tests for the rapid detection of multidrug-resistant Mycobacterium tuberculosis isolates. Mem Inst Oswaldo Cruz 110:649–654. doi:10.1590/0074-02760150136. 26222021 PMC4569829

[19] Campanerut PA, Ghiraldi LD, Spositto FL, Sato DN, Leite CQ, Hirata MH, Hirata RD, Cardoso RF. 2011. Rapid detection of resistance to pyrazinamide in Mycobacterium tuberculosis using the resazurin microtitre assay. J Antimicrob Chemother 66:1044–1046. doi:10.1093/jac/dkr057. 21393185

[20] Martin A, Paasch F, Docx S, Fissette K, Imperiale B, Ribón W, González LA, Werngren J, Engström A, Skenders G, Juréen P, Hoffner S, Del Portillo P, Morcillo N, Palomino JC. 2011. Multicentre laboratory validation of the colorimetric redox indicator (CRI) assay for the rapid detection of extensively drug-resistant (XDR) Mycobacterium tuberculosis. J Antimicrob Chemother 66:827–833. doi:10.1093/jac/dkq527. 21393176

[21] Farnia P, Masjedi MR, Mohammadi F, Tabarsei P, Farnia P, Mohammadzadeh AR, Baghei P, Varahram M, Hoffner S, Velayati AA. 2008. Colorimetric detection of multidrug-resistant or extensively drug-resistant tuberculosis by use of malachite green indicator dye. J Clin Microbiol 46:796–799. doi:10.1128/JCM.01435-07. 18094133 PMC2238113

[22] Mirabal NC, Yzquierdo SL, Lemus D, Madruga M, Milián Y, Echemendía M, Takiff H, Martin A, Van der Stuyf P, Palomino JC, Montoro E. 2010. Evaluation of colorimetric methods using nicotinamide for rapid detection of pyrazinamide resistance in Mycobacterium tuberculosis. J Clin Microbiol 48:2729–2733. doi:10.1128/JCM.00311-10. 20554826 PMC2916567

[23] Rakhmawatie MD, Wibawa T, Lisdiyanti P, Pratiwi WRMustofa. 2019. Evaluation of crystal violet decolorization assay and resazurin microplate assay for antimycobacterial screening. Heliyon 5:e02263. doi:10.1016/j.heliyon.2019.e02263. 31497667 PMC6722264

[24] Palmero DJ. 2007. Tuberculosis and HIV/AIDS, p 570. *In* Palomino JC, Leao SC, Ritacco V. (ed), Tuberculosis: from basic science to patient care, 1st ed, Amedeo Challenge. https://tuberculosistextbook.com/.

[25] Peñuelas-Urquides K, Villarreal-Treviño L, Silva-Ramírez B, Rivadeneyra-Espinoza L, Said-Fernández S, de León MB. 2013. Measuring of Mycobacterium tuberculosis growth. A correlation of the optical measurements with colony forming units. Braz J Microbiol 44:287–289. doi:10.1590/S1517-83822013000100042. 24159318 PMC3804212

[26] Lambrecht RS, Carriere JF, Collins MT. 1988. A model for analyzing growth kinetics of a slowly growing Mycobacterium sp. Appl Environ Microbiol 54:910–916. doi:10.1128/aem.54.4.910-916.1988. 3377502 PMC202572

[27] Hurwitz C, Silverman M. 1949. Measurement of growth of tubercle bacilli by means of a spectrophotometer. Am Rev Tuberc 62:87–90. 10.1164/art.1950.62.1-1.87 15425815

[28] Petroff HA, Steenken W. 1927. Correlation of weight to counting method in determining number of tubercle bacilli. Exp Biol Med 24:958–959. doi:10.3181/00379727-24-3656.

[29] Youmans GP. 1945. The effect of streptomycin in vitro on M. tuberculosis var. Hominis. Q Bull Northwest Univ Med Sch 19:207–209.

[30] Williston EH, Youmans GP. 1949. Factors affecting the sensitivity in vitro of tubercle bacilli to streptomycin. Am Rev Tuberc 59:336–352. 18114721 10.1164/art.1949.59.3.336

[31] Youmans GP, Raleigh GW, Youmans AS. 1947. The tuberculostatic action of para-aminosalicylic acid. J Bacteriol 54:409–416. doi:10.1128/jb.54.4.409-416.1947. 16561376 PMC526571

[32] Kenney M, Johnson PM, Lovelock FJ. 1955. The influence of the size of inoculum on susceptibility testing of Mycobacterium tuberculosis. Am Rev Tuberc 72:390–392. 13249046 10.1164/artpd.1955.72.3.390

[33] Chakraborty S, Rhee KY. 2015. Tuberculosis drug development: history and evolution of the mechanism-based paradigm. Cold Spring Harb Perspect Med 5:a021147. doi:10.1101/cshperspect.a021147. 25877396 PMC4526730

[34] Brook I. 1989. Inoculum effect. Rev Infect Dis 11:361–368. doi:10.1093/clinids/11.3.361. 2664999

[35] Jung YG, Kim H, Lee S, Kim S, Jo E, Kim EG, Choi J, Kim HJ, Yoo J, Lee HJ, Kim H, Jung H, Ryoo S, Kwon S. 2018. A rapid culture system uninfluenced by an inoculum effect increases reliability and convenience for drug susceptibility testing of Mycobacterium tuberculosis. Sci Rep 8:8651. doi:10.1038/s41598-018-26419-z. 29872060 PMC5988837

[36] Madison B, Robinson-Dunn B, George I, Gross W, Lipman H, Metchock B, Sloutsky A, Washabaugh G, Mazurek G, Ridderhof J. 2002. Multicenter evaluation of ethambutol susceptibility testing of Mycobacterium tuberculosis by agar proportion and radiometric methods. J Clin Microbiol 40:3976–3979. doi:10.1128/JCM.40.11.3976-3979.2002. 12409361 PMC139676

